# Inside-Out of Complement in Cancer

**DOI:** 10.3389/fimmu.2022.931273

**Published:** 2022-07-01

**Authors:** Martin Kolev, Madhumita Das, Monica Gerber, Scott Baver, Pascal Deschatelets, Maciej M. Markiewski

**Affiliations:** ^1^ Discovery, Apellis Pharmaceuticals, Waltham, MA, United States; ^2^ Legal Department, Apellis Pharmaceuticals, Waltham, MA, United States; ^3^ Medical Affairs, Apellis Pharmaceuticals, Waltham, MA, United States; ^4^ Department of Immunotherapeutics and Biotechnology, Jerry H. Hodge School of Pharmacy, Texas Tech University Health Sciences Center, Abilene, TX, United States

**Keywords:** cancer, extracellular complement, intracellular complement, biomarkers, therapy, tumor microenvironment, complosome

## Abstract

The role of complement in cancer has received increasing attention over the last decade. Recent studies provide compelling evidence that complement accelerates cancer progression. Despite the pivotal role of complement in fighting microbes, complement seems to suppress antitumor immunity *via* regulation of host cell in the tumor microenvironment. Although most studies link complement in cancer to complement activation in the extracellular space, the discovery of intracellular activation of complement, raises the question: what is the relevance of this process for malignancy? Intracellular activation is pivotal for the survival of immune cells. Therefore, complement can be important for tumor cell survival and growth regardless of the role in immunosuppression. On the other hand, because intracellular complement (the complosome) is indispensable for activation of T cells, these functions will be essential for priming antitumor T cell responses. Here, we review functions of complement in cancer with the consideration of extra and intracellular pathways of complement activation and spatial distribution of complement proteins in tumors and periphery and provide our take on potential significance of complement as biomarker and target for cancer therapy.

## 1 Overview of the Complement System and Its Roles in Immunity

The complement system comprises of over 50 soluble, membrane, and intracellular proteins and is an essential component of both innate and adaptive immunity (1–3). Complement proteins can be activated through proteolytic cleavage in extracellular and intracellular spaces. Therefore, we use terms extracellular and intracellular complement thereafter in reference to these different activation pathways (4). We also consider the terms intracellular complement and the complosome interchangeable (5).

Extracellular activation pathways are well described. They include the classical (CP), lectin (LP), or alternative (AP) pathways (6). Extracellular activation by danger signals, such as bacterial surfaces or immune complexes, results in the opsonization of pathogens and subsequent lysis or removal by phagocytes. Complement activation and proteolytic cleavages of large inactive fragments lead to the generation of smaller size fragments such as C3b, iC3b, C3d, C3a, C5a, and the protein complex MAC (7). The central step in extracellular activation is the cleavage of C3 and C5 with the subsequent generation of C3a and C5a complement anaphylatoxins that are potent regulators of inflammation (2). C5 cleavage by C5 convertase initiates the formation of membrane attack complex (MAC), which directly lyses gram negative bacteria (8). Extracellular complement is redundant. Therefore, its activation is tightly controlled to prevent disease (3). Thus, regulatory mechanisms that serve to limit complement activation to prevent harm to host cells are of great importance for homeostasis. These complement regulators can block complement activation in the fluid phase (complement factor H [CFH] in conjunction with factor I [CFI]) or on cell surface, as is a case with the membrane-bound complement regulators: CD46, CD55, and CD59 (8, 10). The activation and regulation of extracellular complement has recently been reviewed elsewhere (2, 7, 9).

**Figure 1:**
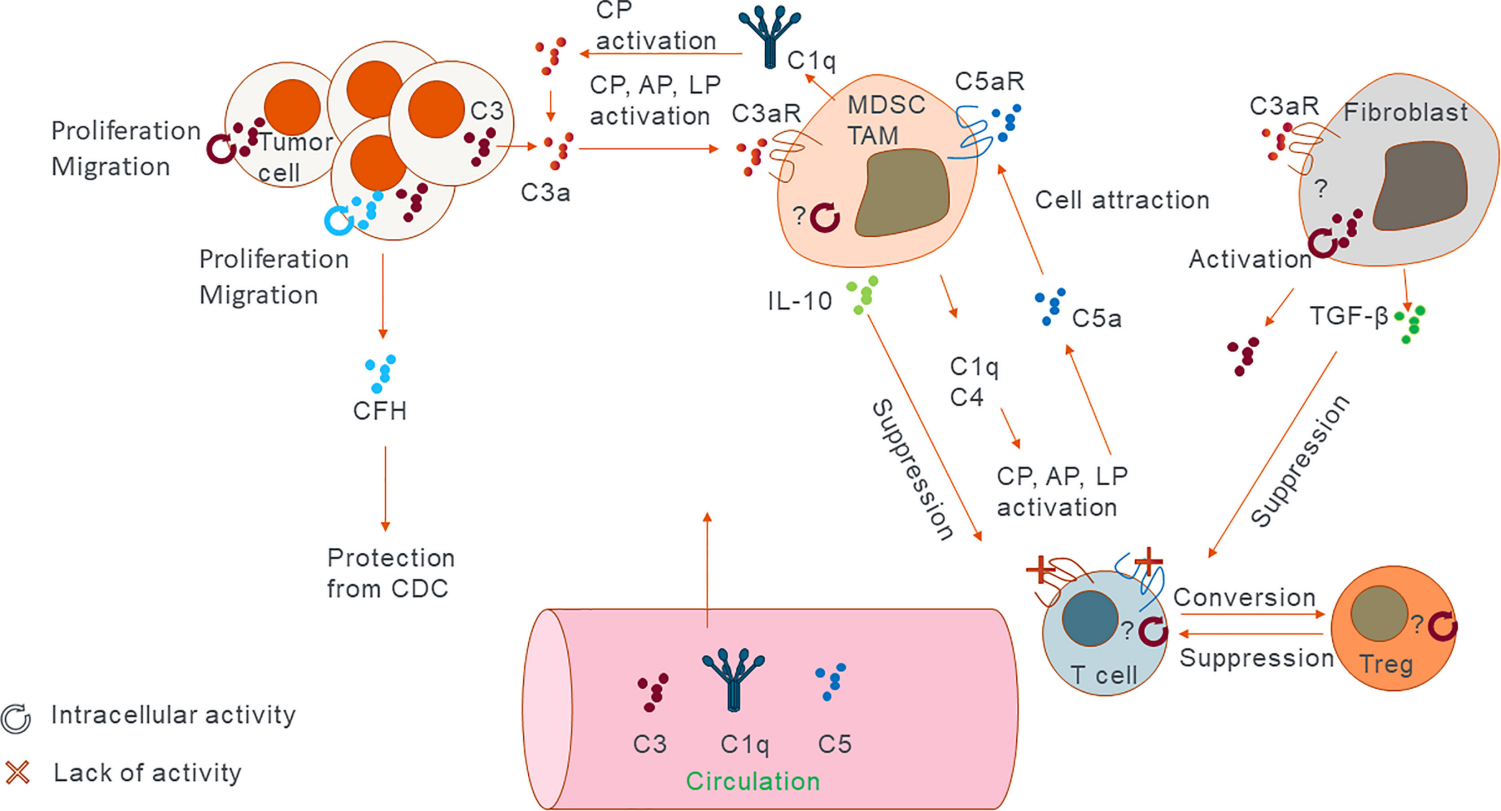
Sites of complement expression and activation. Cartoon representing the main sources of complement in the tumor and their main modes of activation and action. C3 and factor H have shown to exert their function both intra- and extracellularly. Other proteins such as C5 were shown to have extracellular function *via* its receptors. Generally extracellular complement works by inducing immunosuppressive phenotype. Intracellular complement has the potential to modulate immune cell function in similar fashion or by directly increasing proliferation and migration capacity of the cancer cells themselves. In addition, lack of C3aR and C5aR signalling on T cells can convert them into Tregs which in turn can suppress other T cells’ function.

Conversely, intracellular complement, originally described in T cells in 2013, is less understood (11). CD4^+^ T cells were demonstrated to contain intracellular C3 stores that are important for cell survival. Intracellular activation was observed in resting CD4^+^ T cells, where C3 is continuously cleaved by intracellular cathepsin L to bioactive C3a and C3b fragments. C3a then engages with C3aR located on the lysosomal surface, driving mTOR activation and enhancing cell survival (11). Surprisingly, most of intracellular C3 likely derives from serum C3(H_2_O), which is a transient form of C3 (12). T cells also increase expression of C3 upon T cell activation, following the interaction with antigen presenting cells or during the transmigration to the tissues mediated by the LFA-1-AP-1 axis (13). This intrinsic C3 expression in T cells is important, as T cells lacking CD11a (part of LFA-1 heterodimer together with CD18), from the patients with leukocyte adhesion deficiency syndrome, do not upregulate C3 and, therefore, have defective T_H_1responses. During activation, C3 is cleaved intracellularly near the cell membrane and the resulting C3a and C3b fragments are shuttled to the surface where they signal *via* surface expressed C3aR and CD46 receptors, respectively. CD46 plays a crucial role in metabolic reprogramming during T cell activation and enables cell proliferation and cytokine secretion (14). It is therefore not surprising that individuals lacking CD46 expression have reduced T_H_1 immunity (14). Conversely, uncontrolled intracellular C3 activation and CD46 signalling leads to the dysregulation of human T cell responses and contributes to pathologically hyperactive T_H_1 cells in autoimmune disease (e.g., rheumatoid arthritis (RA)) (11, 15). CD46 co-stimulation drives increased expression of the glucose transporter GLUT1 (*SLC2A1*) and the amino acid transporter LAT1 (*SLC7A5*). These increases result in the elevated influx of glucose and amino acids required for T cell activation, proliferation, and the induction of T_H_1 response (14). In addition, CD46 upregulates expression of the Late Endosomal-Lysosomal Adaptor, MAPK and mTOR Activator 5 (LAMTOR5), which is a component of the Ragulator complex sensing amino acid influx. Thus, T cells from CD46-deficient patients do not up-regulate GLUT1, LAT1 and LAMTOR5, do not increase mTOR activity upon activation of T cell receptor, and consequently, do not produce IFN-γ (14). In T cells, CD46 also induces the activation and cleavage of intracellular C5 through yet unknown mechanisms, although intracellular factor B was proposed to play a role in this process in macrophages (16). The resultant C5a engages the intracellular C5aR1 to produce reactive oxygen species and to trigger NLRP3 inflammasome activation, resulting in the expression of intrinsically active IL‐1β, which sustains T_H_1 responses (17). Surface-expressed C5aR2, on the other hand, is a negative regulator of T_H_1 induction. In CD8^+^ T cells, CD46 is required for optimal cytotoxic activity by augmenting nutrient-influx and fatty acid synthesis. However, unlike in CD4^+^ T cells, canonical C5 and NLRP3 inflammasome activity is not required for normal human CD8^+^ T cell activity (18). In mice that normally do not express CD46 in somatic tissue (19), activation of anaphylatoxin receptors C3aR and C5aR1 on T cells may be driving the induction of T_H_1, T_H_17 T cells (20). Additional details on recent advances in intracellular roles of complement in metabolic processes are included in recently published comprehensive reviews (4, 21, 22). Because CD46 plays a crucial role in metabolic changes during T cell activation and metabolic reprograming is critical for cancer cell survival, it is conceivable that similar mechanisms may operate in cancer cells (**Figure 1**) (23).

In summary, the main differences between extra- and intracellular complement include 1) the location of activation process and 2) different overall functions of the complement pathways. Extracellular complement is a danger sensing effector arm of the innate immunity while intracellular complement is probably evolutionary older and is intimately involved in the metabolism and the activation state of innate and adaptive immune cells (24). In addition, intracellular complement activation is not likely to trigger the whole complement cascade until the generation of MAC (C5b-9 complex). While there are suggestions that serum-derived C3 is involved in intracellular activation (12) in T and B cells, recent report suggest that there is a truncated strictly intracellular form of C3 in β-cells in pancreatic islets (25).

An imbalance of activation vs. regulation of both extracellular and intracellular complement results in complement-mediated diseases (1–3, 6, 7). Dysregulation of the extracellular complement system through primary deficiencies in complement regulators results in uncontrolled activation, which leads to archetypical complement-mediated diseases (e.g., paroxysmal nocturnal haemoglobinuria and atypical haemolytic uremic syndrome) (26). Intracellular complement is dysregulated in RA patients where an increase in its activity was observed in synovial CD4^+^ T cells and (11) and fibroblasts (27). These increases seem to correlate with proinflammatory immune cell phenotypes. Inhibition of cathepsin L using cell permeable inhibitor in T cells from RA synovium leads to the reduction in *in vitro* activity (11). Importantly, up to 400 diseases including kidney, ocular, neurodegenerative, and autoimmune disorders, and several cancers are associated with complement dysregulation or alterations in complement genes. However, the functional link between these complement-related alterations and the underlying pathology is often unclear and it is likely that other immune and non-immune mechanisms contribute to these diseases (28–30). An interest in targeting complement for treatment of these disorders has significantly increased over the past twenty years, and particularly following the approval of eculizumab in 2007 and the more recent approval of pegcetacoplan for treatment of paroxysmal nocturnal haemoglobinuria in 2021 (31) and is well summarized in recent reviews (26, 32).

## 2 Overview of Complement in Cancer

Malignancy progression, including growth of primary tumors and cancer metastasis, is regulated by intrinsic mechanisms within cancer cells and crosstalk between tumor cells and elements of the tumor microenvironment, including stromal extracellular matrix proteins, tumor associated fibroblasts, vasculature, and infiltrating immune cells (33). Recent evidence clearly demonstrates a role of complement in orchestrating functions of different components of the tumor microenvironment and that these complement-mediated mechanisms accelerate malignancy progression (8). This notion contrasts the original theory implicating complement in tumor immunosurveillance through complement-mediated cytotoxicity toward cancer cells. While complement activation in solid tumors has been demonstrated (34), there is little evidence that complement can kill a significant number of tumor cells because of evasion mechanisms employed by cancer cells (35). Accordingly, recent preclinical studies demonstrate the role of complement as a tumor promoter (**Figure 1**). The inhibition of antitumor immunity is a main driver of this inflammation-driven acceleration of cancer progression and complement activation seems to be instrumental for orchestrating immunosuppression (36). This has been illustrated in murine models of cancer with targeted deletion of the CFH. This deletion results in excessive complement activation with concomitant increase in tumor burden (37). Genetic analyses of several human cancers reveal high expression of genes encoding proteins of CP and AP, and high expression of some of complement regulators (29). The magnitude of complement activation corresponds to the generation of different levels of complement. Depending on their abundance, effectors may differently impact tumor progression. For example, low sublytic dose of C5b-9 is pivotal for activation of prosurvival signalling in tumor cells (38), whereas a higher dose of C5b-9 has the potential to lyse cells, although tumor cells appear to be resistant to complement-mediated lysis (35). Thus, progressing tumors likely require optimal levels of complement activation that foster chronic inflammation, immunosuppression, angiogenesis, and prosurvival cancer cell signalling (39). C3a and C5a acting through their cognate receptors C3aR, C5aR1 and C5aR2, respectively, are central mediators of tumor promoting responses in the tumor microenvironment (**Figure 1**) (40). In cancer, anaphylatoxins disrupt tissue homeostasis and favour altered immune responses accelerating tumor progression. For example, C5a is a positive modulator of T helper 1 (T_H_1) responses in infection, organ transplantation, and autoimmune diseases (41–43). Conversely, in cancer, C5a inhibits T_H_1 responses and shifts the balance toward T_H_2, which are ineffective in promoting cytolytic antitumor CD8**^+^** T cell activity, or toward regulatory T cells (Tregs) (**Figure 1**). In addition, anaphylatoxins reshape the microenvironment through modulation of myeloid cells. C5a through its interactions with C5aR1 activates and recruits myeloid-derived suppressor cells (MDSC) to tumors and the premetastatic niches and regulates self-renewal of tissue resident pulmonary alveolar macrophages that suppress antitumor immunity (44). The complement system also promotes tumor growth by maintaining stemness of cancer stem cells and promoting tumor proliferation *via* C3a–C3aR signalling (**Figure 1**) (45).

## 3 Complement Dependent Cytotoxicity as Mechanism in Anti-Cancer Therapy

Complement-dependent cytotoxicity (CDC) triggered by MAC is an important mechanism utilized by therapeutic anticancer antibody to kill tumor cells (34). In contrast, antitumor antibodies normally found in patients with cancer are unable to trigger complement-mediated tumor cell killing because of their low quantities and low affinity to tumor antigens (34).

Therapeutic antibodies trigger generation of MAC through the activation of CP, initiated by binding of C1q to immune complexes. AP amplifies this activation, thereby, contributing to CDC. Antibodies currently approved for cancer therapy, with confirmed CDC as part of their effector mechanism of action, target CD20 (rituximab), CD38 [daratumumab, (46)], EGFR [cetuximab (47)], GD2 [dinutuximab, (48)], claudin 18 [zolbetuximab, (49)), and HER2 (pertuzumab) (50)].

These antibodies are of IgG1 isotype, as IgG1 is known to trigger strong complement activation (51). Some of these antibodies have been modified to better activate complement and, consequently, more efficaciously induce tumor cell killing. For example, glycosylation and mannosylation of CD20 mAb obinutuzumab triggers the activation of the lectin pathway, which further potentiates CDC (52). Mutations of the Fc fragment (Glu^345^ to Arg) enables more efficient hexamerization and, consequently, binding of more C1q molecules, which amplifies CP and CDC (53).

The first antibody approved for therapy that utilizes CDC, as part of its therapeutic mechanism, was rituximab (anti-CD20). This antibody is approved for relapsed indolent Non-Hodgkin lymphomas (NHL) and its newer iteration, rituximab with hyaluronidase for subcutaneous injection, was more recently approved for refractory follicular lymphoma, diffuse large B-cell lymphoma (DLBCL), and chronic lymphocytic leukaemia (CLL) (54). Other CD20 antibodies such as ofatumumab and ibritumomab (55), and anti-CD52 (alemtuzumab) were also showed to have complement activating properties and lyse cancer cells through CDC (56).

The therapeutic efficacy of anti-cancer antibodies is, however, hindered by several mechanisms that cancer cells employ to evade complement-mediated lysis. These mechanisms include the removal of MAC from the cell surface (57, 58) and the incorporation of MAC into the cell membrane at sub-lytic concentrations (35). The latter can lead to increased tumorigenesis and faster malignancy progression through increased cancer cell proliferation, enhanced epithelial-to-mesenchymal transition (EMT), and increased motility of cancer cells that all ultimately facilitate metastasis (reviewed in 59). Overexpression of membrane bound complement regulators such as CD46, CD55, and CD 59 and blocking complement activation at the level of C3b also prevent CDC and efficient tumor cell killing. CD46 is overexpressed in breast (60) and ovarian cancers (10), whereas CD59 in sarcomas and melanoma (61). CFH, a fluid phase and cell surface complement regulator, which also reduces CDC, was found in extracellular vesicles (EV) secreted by non-small cell lung carcinoma (NSCLC) (62). In addition, cancer cells evade complement-mediated lysis *via* modulation of the complement regulators through posttranslational modifications. Lin and colleagues showed that ST3GAL1-mediated sialylation of CD55 renders better inhibition of the complement system at C3 level and that ST3GAL1 silencing results in increased C3 deposition and increased CDC-mediated killing of breast cancer cells (63). Interestingly, tumors can produce analogues of complement regulators that have alike inhibitory functions. FH related protein 5 (FHR5) confers resistance of glioblastoma to complement-mediated killing *in vitro* (64). In summary, monoclonal antibodies with CDC, as part of their mechanism of action, are efficacious in haematological malignancies but their efficacy in solid tumors is low (65).

The discovery of complement evasion mechanisms that reduce or completely prevent CDC prompted the attempts to disable complement regulators in tumor cells to increase CDC and, consequently, the efficacy of therapeutic antibodies (34, 66). For example, the inhibition of both CD55 and CD59 results in enhanced CDC and better efficacy of trastuzumab (anti-HER2) in killing human lung carcinoma *in vitro* (67). However, because CD46, CD55, and CD59 are constitutively expressed in human tissues, nonspecific targeting of these molecules can lead to undesired complement activation outside the tumor. In addition to membrane bound complement regulators, targeting soluble complement inhibitors is considered as additional strategy. For instance, the inhibition of CFH, using a recombinant antibody based on the naturally occurring CFH antibodies, was shown to improve rituximab efficacy *in vitro* and *in vivo* (68). Interestingly, CFH antibodies were found in patients with early-stage NSCLC (69).

To avoid adverse effects of non-specific targeting complement regulators, bi-specific antibodies targeting a complement regulator and a tumor antigen were developed. Antibodies targeting CD20 and simultaneously CD55 or CD59 increased the survival of mice in a model of Burkitt’s lymphoma. The improved efficacy of the anti-CD20, largely dependent on CDC, was associated with increased deposition of C3 and C9 in the tumor. The combination of bi-specific anti-CD20/anti-CD55 and anti-CD20/anti-CD59 resulted in near full recovery of treated mice while the treatment with either of those resulted in the survival of only 20% of mice (70). Despite these encouraging results, to the best of our knowledge (clinicalstrials.gov and clinicaltrialsregister.eu), bi-specific antibody therapies, involving inhibition of complement regulators on tumor cells, are not being tested in clinical trials at time of writing this review.

## 4 Sites of Complement Production and Activation

Since the discovery of intracellular complement and associated mechanisms referred to as the complosome (5), the question of how and where to target the complement system for cancer therapy has become more complicated. In the past, the objective was to inhibit complement-mediated mechanisms promoting cancer and enhance CDC for better efficacy of therapeutic antibodies. Now we need to consider multifaced functions of complement inside cells vs. functions of complement effectors in the extracellular space. In addition, since tumor growth relies on the interaction of tumor cells with host-derived microenvironment, it is critically important to address functions of complement inside cancer cells vs. inside host-derived components of the tumor microenvironment. The third dimension of a role of complement in cancer is where complement proteins regulating tumor growth are made. The majority of complement proteins are produced in the liver, in their inactive forms, and are secreted to the extracellular space. Given the rich tumor vasculature, these proteins are readily available in the tumor microenvironment. However, many other cells can produce complement fragments, including immune cells, that are an integral part of the tumor microenvironment. In the tumor, cancer cells, stromal cells, and the immune cells all synthesize and secrete complement proteins (8, 29). Given differences between intracellular vs. extracellular complement functions and diversity of complement sources (**Table 1** and **Figure 1**), it is conceivable that outcome of the regulation of cancer by complement depends on these variables. This has potential implications for future immunotherapies targeting complement.

**Table 1:** Sources, functions, and therapeutic potential of complement proteins in cancer.

Site of Action & Source	Role in Cancer (confirmed or hypothesized)	Therapeutic Potential of Targeting
**Extracellular – Liver-derived**	Promotes cancer progression *via* immune suppression. Kills cancer cells or induce inflammation in presence of therapeutic antibodies.	Small molecule inhibitors and antibodies to target complement receptors or secreted proteins and CDC enhancement
**Extracellular – tumor-derived**	Promotes cancer progression *via* immune suppression.	Small molecule inhibitors and antibodies to target complement receptors or secreted proteins
**Extracellular-non-tumor derived (e.g. stromal cells, immune cells)**	Promotes cancer progression *via* immune suppression.	Small molecule inhibitors and antibodies to target complement receptors or secreted proteins
**Intracellular – immune cell derived**	Potentially, inhibits tumor growth *via* activation of antitumor immunity	Small molecule activators to enhance intracellular complement activation specifically in T cells
**Intracellular – tumor-derived**	Promotes cancer progression *via* activation of prosurvival signalling in tumor cells	Small molecule inhibitors to block intracellular complement activation specifically in tumor cells

### 4.1 Extracellular Complement

#### 4.1.1 Liver-Derived Complement

**Figure 2:**
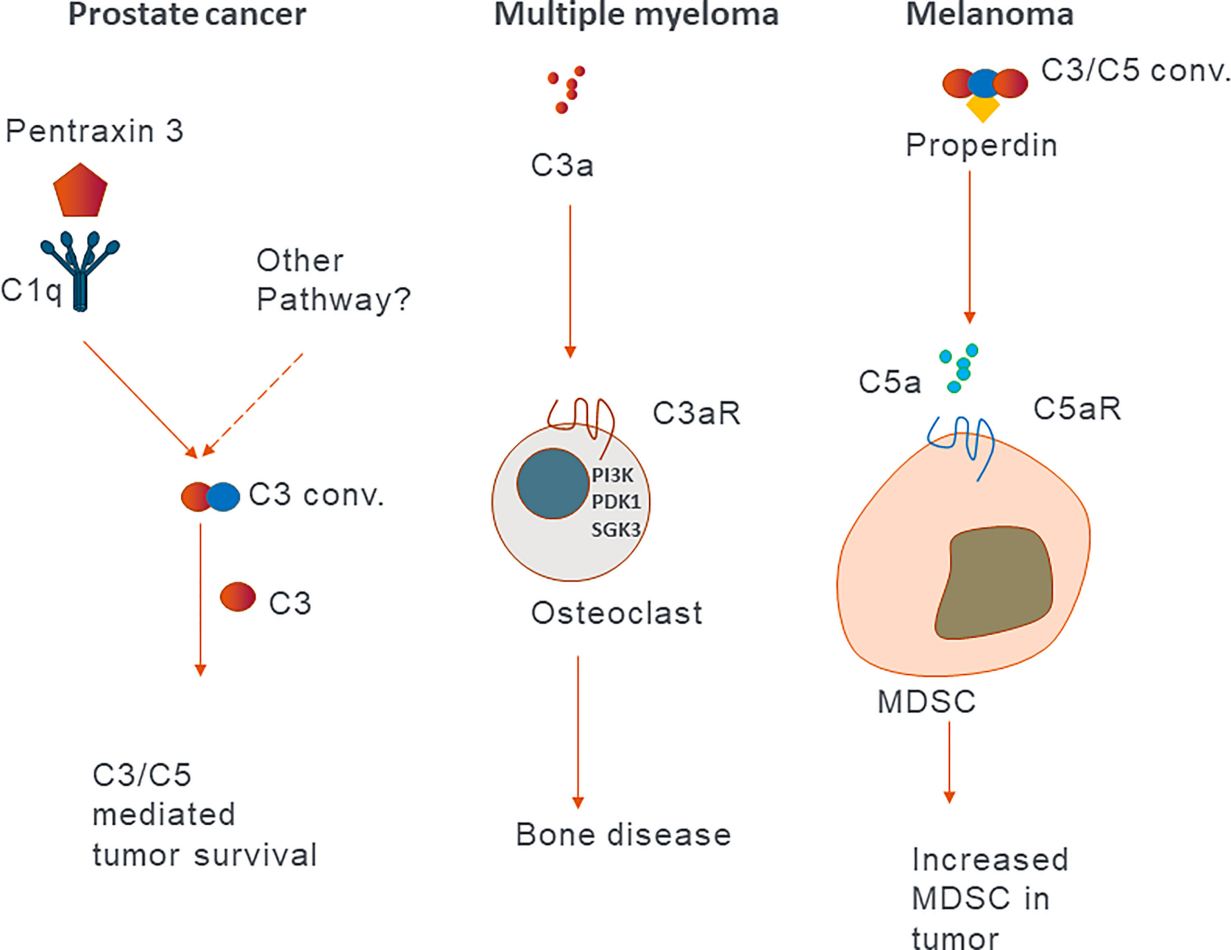
Examples of the effect of extracellular complement in different cancers. Schematic representation of published mechanisms describing different roles of complement proteins in different cancers.

Since the discovery of protumor roles of the C5a-C5aR1 axis in a mouse model of human papilloma virus (HPV)-induced cancer, the view on the role of extracellular complement in cancer has shifted from the concept of complement contribution to tumor immune surveillance to complement mediated suppression of antitumor immunity (71). Here, we will discuss most recent examples of these protumor roles in pre-clinical animal models and human cancers. Extracellular complement activation product C3a induces PI3K/PDK1/p-SGK3 signalling in osteoclasts, which results in bone disease in multiple myeloma patients and is associated with poor prognosis (**Figure 2**) (72). Properdin, which stabilizes the AP C3 convertase, increases the generation of C5a, which *via* its receptor C5aR1, recruits immunosuppressive MDSCs from the spleen in a mouse model of melanoma (73). Similar mechanism, initiated by the activation of CP by pentraxin 3, leads to the recruitment of immunosuppressive cells in prostate cancer patients (74). The C5a-C5aR1 axis also skews the activation of tumor-associated macrophages (TAM) toward M2 anti-inflammatory phenotypes and enables metastasis in a colon cancer mouse model (**Figure 2**) (75). Corroborating mouse data, Piao et al. found that in human colon carcinoma patients, C5aR1 expression correlates with tumor grade. C3 was recently showed to play a role in cutaneous squamous cell carcinoma, as C3 deficient mice developed fewer and smaller tumors compared to wild type controls. Conversely, mice deficient in C5aR1 or C5aR2 had higher tumor incidence, suggesting a tumor limiting role of these receptors in this particular model (76). The protumor roles of C3a-C3aR axis was supported by Magrini and colleagues, who showed that C3 and C3aR deficiencies, in a mouse model of 3-methylcholanthrene sarcomagenesis, result in reduced accumulation and activation of immunosuppressive TAM, which in turn leads to the increased activation of T cells and better response to anti-PD-1 therapy (77). In patients with sarcomas, C3 deficiency-associated genetic signatures predicted better clinical outcomes supporting protumorigenic function of C3. C3a-C3aR contribution to cancer progression was also suggested in a mouse model of pancreatic adenocarcinoma. In this model, the microbiome was found to activate mannose binding lectin (MBL) of LP and promote cancer progression *via* C3 activation because the genetic deletion of either MBL or C3 in the extra tumoral compartment resulted in reduced tumor growth (78). C3, secreted and cleaved extracellularly to C3a and C3b, triggers C3a-C3aR signalling, resulting in increased neutrophil infiltration of tumors in mouse B10 melanoma. These neutrophils were found to promote tumor growth, as their depletion resulted in reduced tumor growth similar to that observed in C3 deficient mice (79). In a mouse renal cell carcinoma (RCC) model, genetic deficiency of C3aR or pharmacological inhibition of either C3aR or C5aR1 resulted in reduced tumor growth. C3aR signalling inhibited antitumor immunity to the greatest extent, as its ablation resulted in highest increase in IFN-γ production by CD8^+^ T cells. Mechanisms of this inhibition involved T cell exhaustion and dysfunction, as C3aR-deficiency and blockade reduced the expression T cell inhibitory receptors Pdcd*1*, *Ctla4*, and *Btla*. Depletion of CD8^+^ T cells entirely abrogated beneficial impact of C3aR-deficiency on tumor growth confirming regulation of T cell function by C3aR (80).

In contrast, the inhibition of C3aR and C5aR1 accelerated tumor growth in a mouse orthotopic head and neck squamous cell carcinoma model. The acceleration of tumor growth associated with the increased number of Tregs in tumors. Mechanistically, locally generated C3a and C5a signalled *via* their receptors on CD4^+^ T cells to promote anti-tumor immune responses. The inhibition of this signalling resulted in the differentiation of CD4^+^ T cells into Tregs. Clearly, Tregs play an important immunosuppressive role in this model, since their depletion resulted in reduced tumor growth (**Figure 1**) (81). The constitutive C3 deficiency did not result in reduction of tumor growth in this model, which further underscores those functions of complement in cancer are highly context and model dependent. Nevertheless, these findings are consistent with the observations that genetic deficiency or pharmacological blockade of C3aR and/or C5aR signalling augments murine and human non-thymic Treg generation and, consequently, reduces the severity of graft-versus-host disease (82). In summary, C3a, C5a, and their receptors appear to have heterogenous functions in cancer and these functions are highly context dependent. Therefore, additional studies involving human cancer cells and patient data are needed to determine the role of liver-derived complement in human cancers.

#### 4.1.2 Tumor Cell-Derived Complement

Complement proteins secreted by tumor cells in the tumor microenvironment and activated extracellularly appear to promote cancer progression similar to liver-derived complement. This notion seems to be valid across several cancer mouse models and human cell lines. C3 secretion induced by Piwi Like RNA-Mediated Gene Silencing 1 protein (PIWIL1) in human hepatocellular carcinoma (HCC) cells leads to the activation of p38 and MAPK signalling in MDSCs, which, in turn, triggers the expression of immunosuppressive cytokine IL-10 in these cells (83). C3 secreted from liver metastatic breast cancer cells was also found to recruit, in C3aR-dependent manner, immature low-density prometastatic neutrophils to the liver (84).

These immunosuppressive and tumor promoting functions of C3 and its cleavage product C3a are contrasted by findings that CFH, which inhibits extracellular complement activation at the level of C3 convertase, promotes cell growth, migration, invasiveness, enhanced liver tumour formation, and metastasis in a mouse HCC model. In line with these observations, treatment with CFH antibody inhibits metastasis in this model (86). The role of complement in tumor immune surveillance is further corroborated by data showing that FHR5, secreted by primary glioblastoma cells, prevented complement activation and lysis of cancer cells *in vitro* (64). However, these two reports are in contrast with the observations that more than 50% of aged male CFH-deficient mice develop spontaneous liver carcinoma. Lack of CFH in these mice correlated with increased complement activation in the liver and increased infiltration of livers with CD8^+^ T cells and macrophages. Accordingly, data from the Cancer Genome Atlas revealed that increased CFH mRNA expression is associated with improved survival in patients with HCC, whereas mutations, reducing CFH function, were associated with worse outcomes. Similar corelative analysis was performed by Daugan et al., who demonstrated that increased CFH mRNA expression is associated with improved survival in patients with RCC (85). While the role of CFH, as an inhibitor of extracellular complement activation, in tumor progression appears to be very much model/context dependent, silencing of intracellular CFH in RCC cell lines results in cell-cycle arrest and death of tumor cells (**Figure 1**) (Daugan, Revel, Thouenon, et al., 2021). In addition to C3 and CFH, cancer cells have been shown to produce and secrete other complement proteins that suppress anti-tumor immunity. RCC secrete complement proteins such as C1r, C1s, C4, and C3 (87). C1r and C1s, secreted by tumor cells, form a functional C1 complex with C1q, secreted by the TAM, to initiate activation of the CP, leading to the generation of immunosuppressive complement effectors (**Figure 1**).

#### 4.1.3 Non-Tumor Cell-Derived Complement

Host cells in the tumor microenvironment, including immune and non-immune cells, such as fibroblasts, can express and secrete C3 and other complement proteins. Functional data for complement proteins derived from these cells are scarce, therefore, we will focus on few examples where the connection to the function has been proposed. Single cell RNA sequencing, broadly adopted by cancer research, has yield ample and detailed data on complement protein expression in solid tumors. Using this approach, Davidson et al. demonstrated the highest expression of C3 in fibroblasts with lower levels in T cells and monocytes in mouse melanoma and breast tumors (88). C3 secreted from these cells is cleaved in the extracellular space and C3a, generated through this cleavage, recruits C3aR expressing macrophages that supress antitumor immunity and accelerate tumor growth. Similar pattern of C3 expression in stromal cells and C3aR in macrophages was found in human melanoma and head and neck tumors. These observations are in line with the earlier work demonstrating that cancer-associated fibroblasts in melanoma can produce C3 and other complement proteins including *C1S*, *C1R*, *C3*, *C4A*, *CFB* and *C1INH* (89). C3 is secreted by hepatic stellate cells in HCC and leads to the expansion of MDSCs and induction of T cell apoptosis *in vitro* (90). Fibroblasts appear to be a major source of C3 and C3a in patients with breast cancer (91). C3a-C3aR signalling in fibroblasts is thought to lead to their activation and subsequent upregulation of pro-tumorigenic cytokines such as transforming growth factor β (TGF-β) and the promotion of lung metastasis *via* EMT, as C3aR deficiency or pharmacological blockade decrease TGF-β expression, EMT, and thus metastasis in a mouse breast cancer model (**Figure 1**). C5 cleavage and activation in the tumor can also be accomplished through non-canonical complement pathways, that do not require the engagement of the entire complement cascade. For example, urokinase positive TAMs can convert plasminogen to plasmin, which directly cleaves C5 to C5a and C5b in C3-independent manner. Then C5a triggers the C5aR1 signalling on tumor-associated mast cell and supresses antitumor CD8^+^ T cells in a mouse model of squamous cell carcinoma (92).

Interestingly, in glioblastoma multiforme (GBM), tumor cells can induce complement protein production in benign host cells through tumor-derived EV. EV contain long noncoding RNA that stimulates C5 and C5a production in microglia. Microglia-derived C5/C5a then promotes the repair of temozolomide (TMZ)-induced DNA damage in C5aR1-dependent manner to confer resistance to TMZ chemotherapy (93). These data were corroborated *in vivo* in an orthotopic mouse model of GBM where C5aR1 antagonism resulted in increased sensitivity of GBM to TMZ. Using *in vitro* co-cultures of breast and prostate cancer cells with lymphatic endothelial cells (LEC), Oliveira-Ferrer demonstrated tumor-dependent induction of C3, CFB and C1q secretion as well as that chemokine upregulation (CCL7, CXCL6, CXCL1) by the LECs promotes tumor metastasis (94).

### 4.2 Intracellular Complement – the Complosome

#### 4.2.1 The Complosome in Tumor Cells

Data on a role of intracellular complement in signalling in tumor cells are limited, recent studies, however, suggest its importance for cancer progression. Cho et al. showed that siRNA downregulation of C3 in ovarian cancer cells SKOV3ip1 leads to reduced tumor cell proliferation and migration, (95). It is conceivable that C3 expressed in tumor cells can be cleaved inside these cells in non-C3 convertase fashion by cathepsins similar to cathepsin L in CD4^+^ T cells (11). Indeed, intracellular C3 cleavage by cathepsin L and B and subsequent release of C3a was demonstrated in colon carcinoma cells (96). Importantly, reduction of C3a secretion by these cells was observed when they were treated with cathepsin L and B inhibitors. These findings are consistent with data showing that C3 produced by mouse colon carcinoma cells is intracellularly activated. This tumor cell-derived C3a promotes TAM activation *via* C3a-C3aR-PI3Kγ pathway leading to the subsequent suppression of antitumor CD8^+^ T cell responses (**Figure 1**) (97). Importantly, the deletion of C3 from the tumor cells enhances the efficacy of anti-PD-L1 therapy in this model (97). Based on these findings, C3, activated in cancer cells, appears to be directly involved in the regulation of cancer cell survival. C3a, generated through this activation and released from cancer cells, contributes to immunosuppression similar to complement effectors from other sources. Supporting this notion, CFB expression was found to be elevated in pancreatic ductal adenocarcinoma (PDAC) (98). CFB mediated protection from senescence of tumor cells and cell proliferation. Secreted from the tumor cells CFB protein correlated with increases of immunosuppressive cells, including Tregs, MDSCs and TAMs. High CFB expression also correlated with poor survival, prompting authors to propose CFB as a potential therapeutic target for PDAC (Shimazaki et al., 2021).

Given abundance of plasma-derived complement, it is not clear if the effects of tumor expressed complement proteins are exclusively related to intracellular complement activity, therefore, further investigation is warranted. since In our opinion the evidence that intracellular complement has specific role in human cancers is still very limited.

Intracellular complement in CD4^+^ T cells and monocytes is involved in the regulation of basic metabolic processes and mTOR during T cell activation (14, 16). Although experimental evidence has yet to come, we theorize that intracellular complement in tumor cells may fulfil similar functions as in T cells. Similarities between cancer cells and activated T cells have been noted, particularly, in the utilization of aerobic glycolysis as means to generate energy and intermediate metabolites that are required for cell survival, proliferation, and metastasis (99). Immunohistochemistry of RCC tumors demonstrated that in the majority of tumors staining for complement was found in infiltrating immune, endothelial, and stromal cells whereas tumor cells did not show positive staining (80) These data are consistent with functions of complement in the regulation of the immunosuppressive microenvironment However, there is a portion of high-grade tumors that exhibited strong cytoplasmic staining for C3. Although evidence supporting the functional link between C3 abundance and tumor differentiation is missing, this notion can be further explored.

#### 4.2.2 The Complosome in Immune and Stromal Cells

C1q directly inhibits the proliferation of T cells (100) and reduces mitochondrial metabolism of CD8^+^ T cells, thereby, restraining their activation (101). Higher expression of C1q and high numbers of the C1q positive macrophages positively correlates with markers of T cell exhaustion (PD-1 and LAG3) and poor clinical outcome for systemic lupus erythematosus (101). Further, C1q^+^ TAMs might be playing similar role in cancer by promoting T cell exhaustion and their tumor accumulation is often associated with poor prognosis (102). C1q is thought to interact with surface bound receptors on TAMs, in addition to controlling T cell metabolism *via* mitochondrial receptor engagement as described by Ling et al. (101). Increased expression of C1q is also associated with reduced survival in patients with RCC (80).

Haldar and colleagues discovered that human prostate carcinoma cells secrete mitochondrial DNA, which triggers TLR9 signalling in fibroblasts. Upon this stimulation, fibroblasts secrete C3a in reactive oxygen species (ROS) dependent manner. Then C3a in a paracrine fashion promotes disease progression and docetaxel resistance by increasing tumor cell proliferation and survival (103). In a subcutaneous xenograft of human PC3 prostate cell line, the authors demonstrated superior efficacy of docetaxel and C3aR inhibition over docetaxel alone in decreasing tumor burden. This was mediated *via* the reduction of AKT and Bcl-2 expression in the tumor cells. However, this paper did not explore in detail if intracellular complement activation occurred, so clearly further studies will be required to elucidate any potential role of intracellular complement in these cells.

## 5 Complement Utility as Biomarker

Complement has been proposed as prognostic or predictive biomarker for many cancers [reviewed in (97)]. The utility of complement in this context seems to be twofold: (1) Complement proteins are routinely measured in plasma and several studies explore the potential of complement in blood to predict prognosis or the response to therapy. (2) Easy access to publicly available genomic data enables analysis of complement gene expression in tumors and correlations with clinical outcomes.

### 5.1 Blood Complement as Biomarker

Recent studies have connected amounts of complement proteins in the blood of cancer patients with clinical outcomes, response to therapy, or diagnosis. The data summarized in **Table 2** paints a picture of increasing number of studies usually identifying an elevation of the concentration of activated complement fragments or complement proteins in plasma or serum of cancer pateints. In most cases this increase correlates with the disease progression, outcome for patients or aids diagnosis. There are however some caveats, including relatively low number of patients and controls in some of the studies and lack of systematic approach to further validate these initial findings. Therefore, larger systematic studies are required in order for complement to be used as biomarker in clinical practice (10).

**Table 2:** Diagnostic, prognostic, and predictive complement biomarkers in blood.

Complement protein	Sample	Expression	Cancer type	Biomarker type	Reference
**C1q and low LDH**	Plasma	Increased	Lung carcinoma	Improved response to ICI	(105)
**C4BP**	Serum	Upregulated in cancer	Pancreatic ductal adenocarcinoma	Diagnosis	(106)
**C3**	Serum	Increased in cancer	Pancreatic ductal adenocarcinoma	Diagnosis	(107)
**C3**	Serum	Increased	Ovarian carcinoma	Poor prognosis	(108)
**C3 and β-2-glycoprotein 1, α-1-acid glycoprotein 2**	Serum	Quantified as panel	Hepatocellular carcinoma	Diagnosis	(109)
**C3f**	Serum	Increased	Metastatic colorectal carcinoma	Diagnosis	(110)
**C3a**	Serum	Increased	Oesophageal carcinoma	Diagnosis	(111)
**C3a-desarg**	Serum	Increased	Breast carcinoma	Diagnosis	(112)
**C3**	Serum	Decreased versus tumor C3	Gastric adenocarcinoma	Poor prognosis	(113)
**MASP-2 and IDH-1**	Serum	Increased in cancer vs healthy	Non-small cell lung carcinoma	Diagnosis	(114)
**C1q**	Serum	Increased in cancer	Glioblastoma Multiforme	Diagnosis	(115)
**CFD**	Serum	Decreased in cancer	Glioblastoma Multiforme	Diagnosis	(115)
**CFB**	Plasma	Increased in cancer	Pancreatic cancer	Diagnosis	(116)
**C4d**	Plasma	Increased levels	Renal cell carcinoma	Poor prognosis	(117)
**C4d**	Plasma, BAL, saliva	Increased levels	Lung cancer	Diagnosis, prognosis	(118, 120; Ajona, Razquin, et al., 2015)
**C4d**	Saliva	Increased levels	Oral squamous cell carcinomas	Associated with end stage	(119)
**FH, FD**	Plasma	Increased	Renal cell carcinoma	Better response to ICI	(80)
**sC5b-9, CFI**	Plasma	Decreased	Renal cell carcinoma	Better response to ICI	(80)
**Both sC5b-9 and C5a**	Plasma	Decreased and increased, respectively	Renal cell carcinoma	Better response to ICI	(80)

### 5.2 Tumor Complement as Biomarker

Complement proteins were shown to be overexpressed in several common human malignancies (121), and their expression are linked with clinical outcomes (29). Predictably, prognostic significance of complement gene expression is dependent on malignancy type. Based on genomic analysis, high expression of *C1QA, C1QB, C1S, C1R, C2, C3, CFB, CFH, CD55*, and *C5aR1* is associated with reduced overall survival in RCC. Conversely, high *CD59* expression is associated with better outcomes (29, 80, 122). Further support for the role of C3 in RCC comes from Dong and colleagues, who demonstrated that high mRNA and protein expression levels of C3 and fibronectin 1 (FN1) in cancer cells were primarily responsible for RCC tumorigenesis and progression (123). Furthermore, increased expression of C3 was also associated with the advanced clinical stage, high pathological grade, and poor survival. The authors suggest both C3 and FN1 are candidate biomarkers for poor patient survival. Increased expression of C3 was also negatively correlated with survival in ovarian carcinoma (124).

In contrast to RCC and ovarian carcinoma, high expression of *C1S, C3, C5, C6*, and *C7* is associated with favourable prognosis in liver carcinoma. *CFB* and *CR2* are also associated with better prognosis in breast cancer alike high expression of *CFD* in pancreatic adenocarcinoma. Upregulated *C3* in colon adenocarcinoma tumor cells was linked to poor overall survival while its expression was lower in rectal adenocarcinoma and had no prognostic value, suggesting context dependent C3 value as a prognostic biomarker (125). *CD59*, which is associated with favourable prognosis in RCC, correlates with poor outcomes in pancreatic, head and neck, and cervical cancer. In addition to RCC, *C5aR1* is associated with poor prognosis in testis and ovarian cancer but with better outcomes in cervical cancer (80). The functional significance of these associations is poorly understood and appear to contradict some mouse studies. For example, increases in the activation of extracellular complement, which generates complement effectors, is expected to trigger immunosuppressive mechanisms that worsen outcomes. Therefore, high expression of complement regulators, which inhibit this activation, is expected to improve outcomes. However, this “rule of thumb” is not universally applicable, as several complement regulators, including *CFH*, *CD55, and CD59*, are associated with poor prognosis in several malignancies (80) For example, increased expression of CD55 in tumor cells correlated with worse survival in stomach and colon adenocarcinomas. Increased C3, complement receptor 4 (CR4), and C5aR1 expression were associated with poor prognosis in gastric cancer patients (125). Finally, C3 expression was found to be higher in primary tumors than lymph node metastases from luminal breast cancer patients (126). The decrease in C3 expression in lymph node metastases was linked to poor prognosis, suggesting that the role of tumor derived C3 might be different in primary tumors vs. metastatic niche and that C3 might play a role in EMT or MET conversion in accordance with earlier studies (127, 128).

Further meta-analyses of the publicly available RNA sequencing or next generation sequencing datasets are required in order to find a link between complement expression in tumor microenvironment and clinical outcomes. Long term this will establish complement as both biomarker and therapeutic target in specific cancers.

## 6 Implications for Therapy

**Figure 3:**
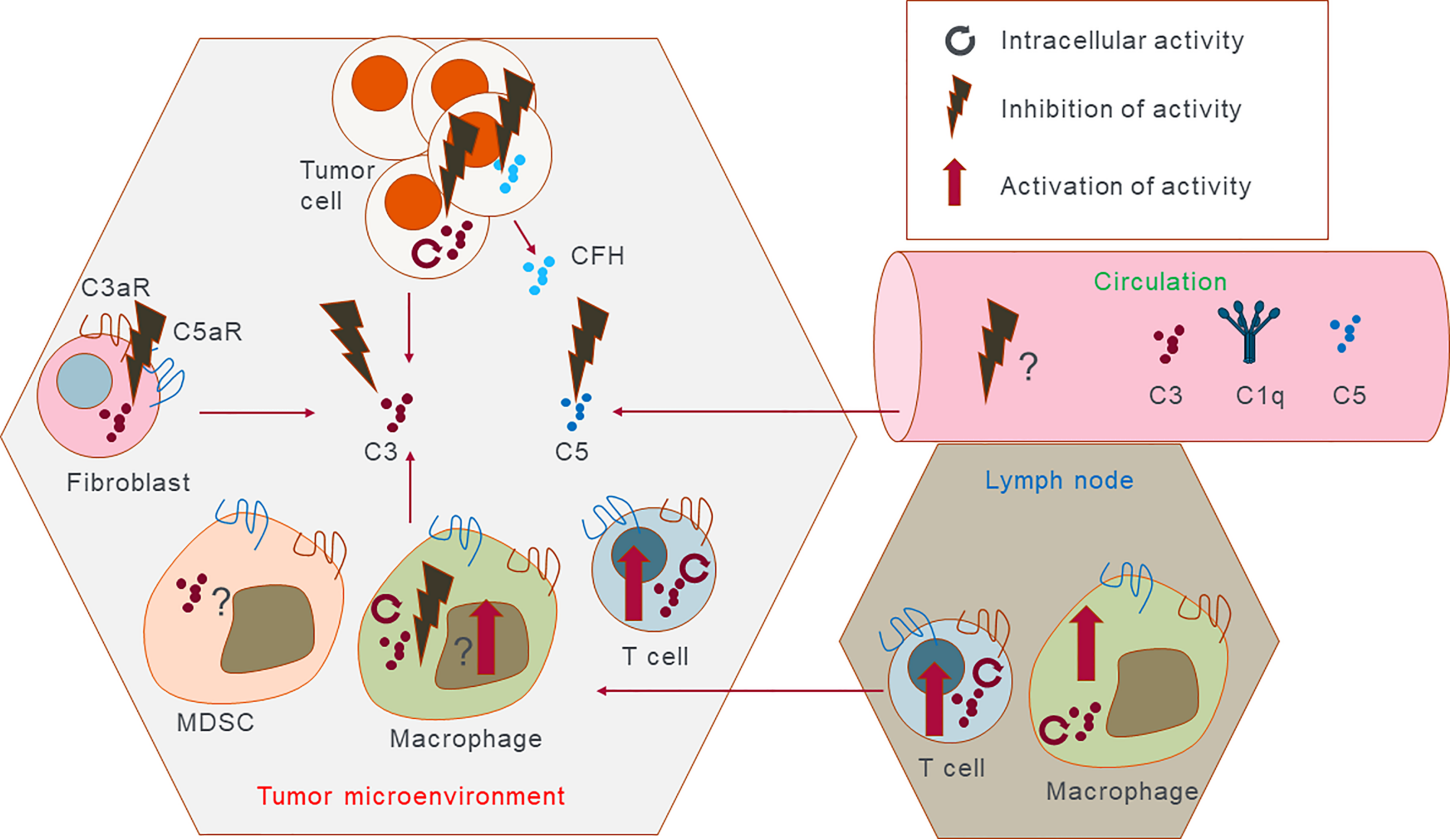
Potential therapeutic intervention points. Complement proteins can be expressed and secreted by almost all cells in the tumor microenvironment, and in the lymph nodes and are present in serum. This cartoon outlines potential targets either intracellularly or extracellularly that could be either inhibited or activated in the case of the immune cells. It is possible that in the future complement inhibiting therapeutics should be able to act extracellularly to promote immune cell antitumor immunity. Reversely intracellular complement in immune cells can be promoted as it is expected to have antitumorigenic effect and promote inflammation. Discreet complement targets present in the tumor cells but not in the immune cells and vice versa would have to be discovered to obtain full clinical efficacy of complement targeting therapy.

Our understanding of the complex role of the complement system in cancer has significantly expanded over the last decade. Initially, complement was viewed as an effector arm to “complement” therapeutic anti-cancer antibodies (such as anti-CD20); however, it is now clear that complement activation, in both the extra- or intracellular space, can directly and indirectly impact tumor growth and metastasis. In addition, complement proteins that influence cancer can be produced not only by ‘traditional’ expressors such as hepatocytes or myeloid cells but also by cancer cells, stromal, and non-myeloid immune cells such as T cells. Correlative analyses linking complement expression in the tumor or blood with clinical outcomes are informative in identifying cancer types where complement may be used as a biomarker, but they still do not address the key question of whether complement functionally drives malignancy progression and poor outcomes. Thus, further pre-clinical mechanistic *in vivo* and *in vitro* research linking complement protein expression and activation in cancer and non-cancers cells with tumor progression and metastasis is needed to gain confidence that targeting complement will be beneficial for cancer patients. Currently, one of the biggest weaknesses of available data is that they are mostly derived from animal models and from constitutive complement knockouts. The clinical translation of these findings is not always straightforward (129). Nevertheless, testing of complement inhibition is now underway in the clinic with both C3 and C5aR1 as targets. IPH5401 (anti-C5aR antibody) was tested in a clinical trial together with anti-PD-L1 in patients with advanced solid tumors. However at the time of writing this review no results have been posted (see **Table 3**). Two other clinical trials are currently active in testing the anti-C5aR monoclonal antibody TJ210001 as monotherapy. C3 extracellular inhibition is also being tested in the clinic in ovarian cancers in combination with Pembrolizumab or Bevacizumab and Pembrolizumab (see **Table 3** for further details). The data obtained from these studies will inform further developments for complement-based cancer therapy and could validate extracellular complement as a target in oncology. Emerging data that intracellular complement might have a role in cancer cell proliferation, survival, and metastasis will require further preclinical validation to gain more confidence that this pathway plays a role in cancer and is targetable for therapy. Since this pathway is operative in immune cells as well, a cancer specific intracellular complement targeting may be required to achieve optimal efficacy (outlined in **Figure 3**).

**Table 3:** Complement therapeutics currently tested in clinical trials.

Drug	Sponsor	Target	Modality	Cancer type	Therapy	Stage	Study Identifier
IPH5401	Innate Pharma	C5aR1	Antibody	Advanced solid tumors	Combinational with durvalumab (anti-PD-L1)	Ph1, Terminated	NCT03665129
TJ210001	I-Mab Biopharma	C5aR1	Antibody	Advanced solid tumors	Monotherapy	Ph1, ongoing	NCT04947033
TJ210001	I-Mab Biopharma	C5aR1	Antibody	Solid Tumor Metastatic Cancer Advanced Cancer	Monotherapy	Ph1, ongoing	NCT04678921
APL-2, pegcetacoplan	Apellis Pharmaceuticals	C3, C3b, C3c	Pegylated peptide	Various adenocarcinomas and carcinomas	Combinational with Bevacizumab (anti-VEGF) and Pembrolizumab (anti-PD-1)	Ph2, ongoing	NCT04919629

Interactions between cancer cells, immune, and non-immune cells in the tumor microenvironment as well as the spatial distribution of complement proteins add complexity to multifaced roles of complement in cancer and present a significant challenge to develop therapy. Ongoing clinical trials and pre-clinical efforts in targeting complement as standalone or combinational therapy will bring a wealth of new information to identify complement targets for tumor specific and cell specific basis and thus enable the design of a new generation of complement therapeutics to harness the full potential of this system for cancer immunotherapy. Furthermore, with the discovery of CRIPSR/Cas9 editing technology and recent advances in AAV and siRNA therapy, options of how-to best target complement in the extra- and intracellular space have never been better.

## Author Contributions

The idea of the manuscript was conceived by MK and MM. All authors contributed to writing, editing and revising of the paper. All authors contributed to the article and approved the submitted version.

## Conflict of Interest

MK, MD, SB, MG and PD are employees and shareholders in Apellis Pharmaceuticals Inc., which actively develops drugs targeting the complement system.

The remaining author declares that the research was conducted in the absence of any commercial or financial relationships that could be construed as a potential conflict of interest.

## Publisher’s Note

All claims expressed in this article are solely those of the authors and do not necessarily represent those of their affiliated organizations, or those of the publisher, the editors and the reviewers. Any product that may be evaluated in this article, or claim that may be made by its manufacturer, is not guaranteed or endorsed by the publisher.
